# Racial/Ethnic Disparities in Pregnancy-Related Deaths — United States, 2007–2016

**DOI:** 10.15585/mmwr.mm6835a3

**Published:** 2019-09-06

**Authors:** Emily E. Petersen, Nicole L. Davis, David Goodman, Shanna Cox, Carla Syverson, Kristi Seed, Carrie Shapiro-Mendoza, William M. Callaghan, Wanda Barfield

**Affiliations:** ^1^Division of Reproductive Health, National Center for Chronic Disease Prevention and Health Promotion, CDC; ^2^DB Consulting Group, Atlanta, Georgia.

Approximately 700 women die in the United States each year as a result of pregnancy or its complications, and significant racial/ethnic disparities in pregnancy-related mortality exist (*1*). Data from CDC’s Pregnancy Mortality Surveillance System (PMSS) for 2007–2016 were analyzed. Pregnancy-related mortality ratios (PRMRs) (i.e., pregnancy-related deaths per 100,000 live births) were analyzed by demographic characteristics and state PRMR tertiles (i.e., states with lowest, middle, and highest PRMR); cause-specific proportionate mortality by race/ethnicity also was calculated. Over the period analyzed, the U.S. overall PRMR was 16.7 pregnancy-related deaths per 100,000 births. Non-Hispanic black (black) and non-Hispanic American Indian/Alaska Native (AI/AN) women experienced higher PRMRs (40.8 and 29.7, respectively) than did all other racial/ethnic groups. This disparity persisted over time and across age groups. The PRMR for black and AI/AN women aged ≥30 years was approximately four to five times that for their white counterparts. PRMRs for black and AI/AN women with at least some college education were higher than those for all other racial/ethnic groups with less than a high school diploma. Among state PRMR tertiles, the PRMRs for black and AI/AN women were 2.8–3.3 and 1.7–3.3 times as high, respectively, as those for non-Hispanic white (white) women. Significant differences in cause-specific proportionate mortality were observed among racial/ethnic populations. Strategies to address racial/ethnic disparities in pregnancy-related deaths, including improving women’s health and access to quality care in the preconception, pregnancy, and postpartum periods, can be implemented through coordination at the community, health facility, patient, provider, and system levels.

PMSS was established in 1986 by CDC and the American College of Obstetricians and Gynecologists to better understand the causes of death and risk factors associated with pregnancy-related deaths. Methodology of PMSS has been described previously (*2*). Briefly, CDC requests that all states, the District of Columbia, and New York City identify deaths during or within 1 year of pregnancy and send corresponding death certificates, linked birth or fetal death certificates, and additional data when available. Medically trained epidemiologists review information and determine the relatedness to pregnancy and cause for each death. A death was considered pregnancy-related if it occurred during or within 1 year of pregnancy and was caused by a pregnancy complication, a chain of events initiated by pregnancy, or aggravation of an unrelated condition by the physiologic effects of pregnancy. U.S. natality files were the source of live birth data (*3*).

PRMRs were analyzed by age group, highest level of education, and calendar year for women who were non-Hispanic white, black, AI/AN, Asian or Pacific Islander (A/PI), and Hispanic. Per the PMSS assurance of confidentiality, state-specific data are not authorized to be released. States were anonymously classified by PRMR and grouped into lowest, middle, and highest tertiles by PRMR; the PRMR was calculated by race/ethnicity per state tertile. Disparity ratios (comparisons of PRMR between two racial/ethnic groups) were calculated by five 2-year intervals, demographic characteristics, and state PRMR tertiles. White decedents were the referent group because they represented the largest racial/ethnic group. Cause-specific proportionate mortality was classified in 10 mutually exclusive categories,* and differences by race/ethnicity were identified using chi-squared tests. SAS statistical software (version 9.4; SAS Institute) was used for the analyses.

During 2007–2016, a total of 6,765 pregnancy-related deaths occurred in the United States (PRMR = 16.7 per 100,000 births). PRMRs were highest among black (40.8) and AI/AN (29.7) women; these rates were 3.2 and 2.3 times the PRMR for white women (12.7) (Table 1). From 2007–2008 to 2015–2016, the overall PRMR increased slightly from 15.0 to 17.0. The disparity ratios did not change significantly over time.

**TABLE 1:** Pregnancy-related mortality ratios (PRMRs) (pregnancy-related deaths per 100,000 live births) and disparity ratios by age group, education, tertile of states, and race/ethnicity* — United States, 2007–2016^†^

Characteristic	Total PRMR	White PRMR	Black PRMR	Black: white disp. ratio	AI/AN PRMR	AI/AN: white disp. ratio	A/PI PRMR	A/PI: white disp. ratio	Hispanic PRMR	Hispanic: white disp. ratio
**Total**	**16.7**	**12.7**	**40.8**	**3.2**	**29.7**	**2.3**	**13.5**	**1.1**	**11.5**	**0.9**
**Age group (yrs)**										
<20	10.9	10.8	16.8	1.5	19.5	1.8	—^§^	—	6.7	0.6
20–24	12.2	9.6	26.3	2.7	11.6	1.2	7.2	0.7	7.0	0.7
25–29	13.3	9.3	37.0	4.0	25.2	2.7	9.5	1.0	9.6	1.0
30–34	15.8	11.3	48.6	4.3	41.2	3.7	12.5	1.1	12.6	1.1
35–39	27.7	20.5	80.7	3.9	104.2	5.1	18.8	0.9	22.6	1.1
≥40	65.2	51.5	189.7	3.7	—	—	36.6	0.7	44.0	0.9
**Education completed**										
Less than high school	21.6	25.0	45.6	1.8	50.8	2.0	18.7	0.7	12.6	0.5
High school	27.4	25.2	59.1	2.3	43.7	1.7	22.9	0.9	11.2	0.4
Some college	16.4	11.7	41.0	3.5	32.0	2.7	15.4	1.3	9.4	0.8
College graduate or higher	10.9	7.8	40.2	5.2	—	—	13.2	1.7	9.3	1.2
**Period**										
2007–2008	15.0	11.5	35.6	3.1	26.9	2.3	11.4	1.0	10.8	0.9
2009–2010	17.3	12.8	41.6	3.2	30.7	2.4	13.6	1.1	12.8	1.0
2011–2012	16.8	12.4	44.3	3.6	38.4	3.1	11.6	0.9	10.4	0.8
2013–2014	17.6	13.5	42.1	3.1	30.3	2.2	15.8	1.2	12.0	0.9
2015–2016	17.0	13.2	40.8	3.1	21.9	1.7	14.7	1.1	11.6	0.9
**State-level PRMR tertile**										
Lowest PRMR	10.7	8.7	26.0	3.0	28.9	3.3	11.9	1.4	9.7	1.1
Middle PRMR	15.4	11.0	36.9	3.3	33.9	3.1	14.2	1.3	11.7	1.1
Highest PRMR	21.9	16.6	45.9	2.8	28.8	1.7	15.8	0.9	13.2	0.8

**Abbreviations:** AI/AN = American Indian/Alaska Native; A/PI = Asian/Pacific Islander.

* Blacks, whites, AI/AN, and A/PI were non-Hispanic; Hispanic women might be of any race.

^†^ 25 pregnancy-related deaths with unknown race/ethnicity were included in the total analyses but not presented in an individual column; two pregnancy-related deaths with unknown age were excluded from age analyses; 687 pregnancy-related deaths with unknown educational levels were excluded from education analyses.

^§^ Dashes indicate fewer than 10 deaths; these results were suppressed because ratios might be unreliable.

PRMR increased with maternal age; the black:white disparity was lowest among women aged <20 years (1.5) and highest among those aged 30–34 years (4.3); the AI/AN:white disparity was lowest among women aged 20–24 years (1.2) and was highest among women aged 35–39 years (5.1). Racial/ethnic disparities were present at all education levels. The PRMR among black women with a completed college education or higher was 1.6 times that of white women with less than a high school diploma. Among women with a college education or higher, the PRMR for black women was 5.2 times that of their white counterparts. The black:white disparity ratio in the PRMR for the states in the lowest, middle, and highest tertiles was 3.0, 3.3, and 2.8, respectively.

Cardiovascular conditions (including cardiomyopathy, other cardiovascular conditions, and cerebrovascular accidents), other noncardiovascular medical conditions, and infection were leading causes of pregnancy-related deaths. The proportion of pregnancy-related deaths attributed to each of 10 mutually exclusive causes varied by race/ethnicity (Table 2). Cardiomyopathy, thrombotic pulmonary embolism, and hypertensive disorders of pregnancy contributed to a significantly higher proportion of pregnancy-related deaths among black women than among white women. Hemorrhage and hypertensive disorders of pregnancy contributed to a higher proportion of pregnancy-related deaths among AI/AN women than among white women.

**TABLE 2:** Cause-specific pregnancy-related mortality, by race/ethnicity — Pregnancy Mortality Surveillance System, United States, 2007–2016

Cause of death	Proportionate cause of death by race/ethnicity* No. (%) attributed to each cause					Total deaths
	White	Black	AI/AN	A/PI	Hispanic	
Hemorrhage	250 (9.1)	237 (9.7)	23 (19.7)^†^	66 (19.5)^†^	173 (15.8)^†^	**752 (11.1)**
Infection	418 (15.2)	235 (9.7)^§^	10 (8.5)^§^	51 (15.0)	183 (16.7)	**900 (13.3)**
Amniotic fluid embolism	147 (5.3)	106 (4.4)	3 (2.6)	51 (15.0)^†^	58 (5.3)	**365 (5.4)**
Thrombotic pulmonary or other embolism	246 (8.9)	265 (10.9)^†^	9 (7.7)	11 (3.2)^§^	88 (8.0)	**624 (9.2)**
Hypertensive disorders of pregnancy	184 (6.7)	200 (8.2)^†^	15 (12.8)^†^	21 (6.2)	106 (9.7)^†^	**528 (7.8)**
Anesthesia complications	7 (0.3)	14 (0.6)	0 (0.0)	3 (0.9)	6 (0.5)	**30 (0.4)**
Cerebrovascular accidents	207 (7.5)	148 (6.1)^§^	6 (5.1)	37 (10.9)^†^	92 (8.4)	**490 (7.2)**
Cardiomyopathy	288 (10.4)	345 (14.2)^†^	17 (14.5)	21 (6.2)^§^	75 (6.8)^§^	**748 (11.1)**
Other cardiovascular conditions	465 (16.9)	393 (16.2)	13 (11.1)	38 (11.2)^§^	124 (11.3)^§^	**1,035 (15.3)**
Other noncardiovascular medical conditions	384 (13.9)	343 (14.1)	16 (13.7)	26 (7.7)^§^	130 (11.9)	**903 (13.3)**
Unknown	160 (5.8)	146 (6.0)	5 (4.3)	14 (4.1)	61 (5.6)	**390 (5.8)**
**Total**	**2,756**	**2,432**	**117**	**339**	**1,096**	**6,765^¶^**

**Abbreviations:** AI/AN = American Indian/Alaska Native; A/PI = Asian/Pacific Islander.

* Black, white, AI/AN, and A/PI women were non-Hispanic; Hispanic women could be of any race.

^†^ Significantly higher proportion of pregnancy-related deaths compared with that among white women, p<0.05.

^§^ Significantly lower proportion of pregnancy-related deaths compared with that among white women, p<0.05.

^¶^ Twenty-five pregnancy-related deaths with unknown race/ethnicity were included in the total but not elsewhere in the table.

## Discussion

Racial/ethnic disparities in pregnancy-related mortality were evident in 2007 and continued through 2016, with significantly higher PRMRs among black and AI/AN women than among white, A/PI, and Hispanic women. The PRMR for black and AI/AN women aged ≥30 years was approximately four to five times that of their white counterparts. Even in states with the lowest PRMRs, and among groups with higher levels of education, significant disparities persisted, demonstrating that the disparity in pregnancy-related mortality for black and AI/AN women is a complex national problem.

Multiple factors contribute to pregnancy-related mortality and to racial/ethnic disparities. Previous analyses found that for each pregnancy-related death, an average of three to four contributing factors were identified at multiple levels, including community, health facility, patient/family, provider, and system (*1*). Thirteen state maternal mortality review committees reported 60% of pregnancy-related deaths were preventable, and there were no significant differences in preventability by race/ethnicity (*1*). Differences in proportionate causes of death among black and AI/AN women might reflect differences in access to care, quality of care, and prevalence of chronic diseases (*4*).

Chronic diseases associated with increased risk for pregnancy-related mortality (e.g., hypertension) are more prevalent and less well controlled in black women (*5*). Ensuring access to quality care, including specialist providers, during preconception, pregnancy, and the postpartum period is crucial for all women to identify and manage chronic medical conditions (*4*). Systemic factors (e.g., gaps in health care coverage and preventive care, lack of coordinated health care, and social services) and community factors (e.g., securing transportation for medical visits and inadequate housing) have also been identified as contributors to pregnancy-related deaths (*1*). Addressing these factors and ensuring that pregnant women at high risk for complications receive care in facilities prepared to provide the required level of specialized care can improve outcomes.^†^^,^^§ ^In addition, innovative delivery of care models in the preconception, pregnancy, and postpartum periods might be further evaluated for their potential to reduce maternal disparities.

Quality of care likely has a role in pregnancy-related deaths and associated racial disparities. A national study of five specific pregnancy complications found a similar prevalence of complications among black and white women, but a significantly higher case-fatality rate among black women (*6*). Studies have suggested that black women are more likely than are white women to receive obstetric care in hospitals that provide lower quality of care (*7*). Hospitals and health care systems can implement standardized protocols and training in quality improvement initiatives, ensuring implementation in facilities that serve disproportionately affected communities. Quality improvement efforts, such as perinatal quality collaboratives^¶^ that facilitate a change in the culture of care provision, implement standards of care,** and rapidly use data to identity opportunities for improvement, can improve the quality of care received by all pregnant and postpartum women.

Implicit racial bias has been reported in the health care system and can affect patient-provider interactions, treatment decisions, patient adherence to recommendations, and patient health outcomes (*8*). This report’s findings demonstrate that black and AI/AN women have a more accelerated trajectory in age-specific PRMRs compared with white women. This might be related to the “weathering” hypothesis, which proposed that black women experience earlier deterioration of health because of the cumulative impact of exposure to psychosocial, economic, and environmental stressors (*9*). Identifying and addressing implicit bias and structural racism in health care and community settings, engaging communities in prevention efforts, and supporting community-based programs that build social support and resiliency would likely improve patient-provider interactions, health communication, and health outcomes (*4*).

Reducing disparities in pregnancy-related mortality requires addressing multifaceted contributors. Ensuring robust comprehensive data collection and analysis through state and local maternal mortality review committees, which thoroughly review pregnancy-related deaths and make actionable prevention recommendations, offer the best opportunity for identifying priority strategies to reduce disparities in pregnancy-related mortality.^††^

The findings in this report are subject to at least three limitations. First, PMSS predominantly uses death certificates and linked birth or fetal death certificates to determine the pregnancy-relatedness of each death. Errors in reported pregnancy status on death certificates have been described, potentially leading to overestimation of the number of pregnancy-related deaths (*10*). Second, pregnancy-relatedness cannot generally be determined in PMSS for cancer-related deaths or injury deaths such as drug overdoses, suicides, or homicides, and thus, these are often not included in the PRMR calculated from PMSS data. Finally, small cohort sizes precluded the reporting of some factors by race/ethnicity; in addition, there might be inconsistencies in the reporting of race/ethnicity when death certificates were used for classification.^§§^

Most pregnancy-related deaths can be prevented, and significant racial/ethnic disparities in pregnancy-related mortality need to be addressed. Further identification and evaluation of factors contributing to racial/ethnic disparities are crucial to inform and implement prevention strategies that will effectively reduce disparities in pregnancy-related mortality, including strategies to improve women’s health and access to quality care in the preconception, pregnancy, and postpartum periods. Addressing this complex national problem requires coordination and collaboration among community organizations, health facilities, patients and families, health care providers, and health systems.

**Summary**

**What is already known about this topic?**

Approximately 700 women die annually in the United States as a result of pregnancy or its complications; racial/ethnic disparities exist.

**What is added by this report?**

During 2007–2016, black and American Indian/Alaska Native women had significantly more pregnancy-related deaths per 100,000 births than did white, Hispanic, and Asian/Pacific Islander women. Disparities persisted over time and across age groups and were present even in states with the lowest pregnancy-related mortality ratios and among groups with higher levels of education. The cause-specific proportion of pregnancy-related deaths varied by race/ethnicity.

**What are the implications for public health practice?**

Identifying factors that drive differences in pregnancy-related deaths and implementing prevention strategies to address them could reduce racial/ethnic disparities in pregnancy-related mortality. Strategies to address racial/ethnic disparities in pregnancy-related deaths, including improving women’s health and access to quality care in the preconception, pregnancy, and postpartum periods, can be implemented through coordination at the community, health facility, patient and family, health care provider, and system levels.

## Footnotes

* Cause of death coding includes the following 10 mutually exclusive categories: hemorrhage; infection; amniotic fluid embolism; thrombotic pulmonary or other embolism (i.e., air, septic, or fat); hypertensive disorders of pregnancy (i.e., preeclampsia or eclampsia); anesthesia complications; cerebrovascular accidents; cardiomyopathy; other cardiovascular conditions (e.g., congenital heart disease, ischemic heart disease, cardiac valvular disease, hypertensive heart disease, and congestive heart failure); and other noncardiovascular medical conditions (e.g., endocrine, hematologic, immunologic, and renal). Deaths caused by hypertension that were not preeclampsia, eclampsia, or gestational hypertension were categorized in the “other cardiovascular conditions” category. Deaths caused by cerebrovascular accidents that were a result of preeclampsia or eclampsia were classified in the “hypertensive disorders of pregnancy” category; otherwise, deaths were classified in the “cerebrovascular accidents” category.

† https://www.cdc.gov/reproductivehealth/maternalinfanthealth/cdc-locate/index.html.

§ https://www.acog.org/About-ACOG/ACOG-Departments/LOMC?IsMobileSet=false.

¶ https://www.cdc.gov/reproductivehealth/maternalinfanthealth/pqc.htm.

** https://safehealthcareforeverywoman.org/patient-safety-bundles/.

§§ Whenever possible, data for race and Hispanic origin were obtained from linked birth certificates, which are self-reported; however, when not available, data for specified race or Hispanic-origin were obtained from death certificates.
